# Tissue-specific variability in protein expression of cytochrome P450 and glutathione S-transferase isoenzymes in non-small cell lung carcinoma

**DOI:** 10.2478/aiht-2026-77-4112

**Published:** 2026-09-25

**Authors:** Murat Kılıç, Serpil Oğuztüzün, Sezgin Çelik, Funda Demirağ, Pınar Bıçakçıoğlu, Mümtaz İşcan, Ahmet Oğuz Ada

**Affiliations:** Ankara University Vocational School of Health Services, Department of Pharmacy Services, Ankara, Turkey; Kırıkkale University Faculty of Arts and Sciences, Department of Biology, Kırıkkale, Turkey; Yıldız Technical University Faculty of Arts and Sciences, Department of Molecular Biology and Genetics, İstanbul, Turkey; University of Health Sciences, Ankara Atatürk Sanatorium Training and Research Hospital, Department of Pathology, Ankara, Turkey; University of Health Sciences, Ankara Atatürk Sanatorium Training and Research Hospital, Department of Thoracic Surgery, Ankara, Turkey; Cyprus International University Faculty of Pharmacy, Department of Pharmaceutical Toxicology, Nicosia, Northern Cyprus; Ankara University Faculty of Pharmacy, Department of Pharmaceutical Toxicology, Ankara, Turkey

**Keywords:** adenocarcinoma, biotransformation, carcinogenesis, CYP1A1, CYP1B1, CYP2E1, GSTM1, GSTP1, GSTT1, immunohistochemistry, lung neoplasms, squamous cell carcinoma, xenobiotics, adenokarcinom, biotransformacija, CYP1A1, CYP1B1, CYP2E1, glutation S-transferaza M1 (GSTM1), glutation S-transferaza P1 (GSTP1), glutation S-transferaza T1 (GSTT1), imunohistokemija, kancerogeneza, karcinom nemalih stanica pluća, ksenobiotici, novotvorine pluća, planocelularni karcinom

## Abstract

Earlier tissue-based studies report heterogeneous CYP and GST protein expression in non-small cell lung cancer (NSCLC), but simultaneous assessment of phase I and II enzymes in paired tumour and peripheral normal tissues remains limited and their expression patterns poorly characterised across the two major NSCLC histological subtypes, namely adenocarcinoma (AC) and squamous cell carcinoma (SCC). To address these gaps, this retrospective study evaluated CYP1A1, CYP1B1, CYP2E1, GSTM1, GSTT1, and GSTP1 expression immunohistochemically in tumour and peripheral normal lung tissues of 50 patients with NSCLC, 27 of whom with AC and 23 with SCC. Cytoplasmic staining intensity was scored from 0 to 3. Tissue and subtype comparisons were performed using the Mann-Whitney *U* test, and associations with clinical variables were assessed using Spearman's rank correlation. CYP2E1 expression was significantly higher in tumour than in normal tissue in the combined NSCLC group and in both histological subtypes (p<0.05). CYP1A1 expression was higher in AC tumours than in normal tissue and SCC tumours (both p<0.05). GSTM1 expression was higher in tumour tissue in the combined NSCLC group and in the SCC subgroup and was also higher in SCC than in AC tumours (p<0.05). GSTT1 expression was higher in AC than SCC tumours (p<0.05). CYP1B1 and GSTP1 were highly expressed in both tissue compartments but showed no significant tumour-specific increase. CYP and GST isoenzymes therefore showed tissue- and subtype-dependent rather than uniform expression patterns in NSCLC. The differences observed in CYP2E1, CYP1A1, GSTM1, and GSTT1 suggest that AC and SCC may differ in xenobiotic metabolism and cellular defence, and that these isoenzymes may contribute to metabolic processes associated with NSCLC pathogenesis.

## Abstract (hr)

Ranija istraživanja na tkivima upućuju na heterogenu ekspresiju proteina CYP i GST kod karcinoma nemalih stanica pluća (engl. *non-small cell lung cancer*, krat. NSCLC), no i dalje se malo zna o njihovoj ekspresiji u stadijima 1 i 2 u usporednim uzorcima tumora i perifernog zdravog tkiva. Također su slabo karakterizirani njihovi obrasci ekspresije u dvama glavnim histološkim podtipovima NSCLC-a: adenokarcinomu (AC) i planocelularnom karcinomu (SCC). Stoga smo u ovom retrospektivnom istraživanju imunohistokemijski ocijenili ekspresiju CYP1A1, CYP1B1, CYP2E1, GSTM1, GSTT1 i GSTP1 u tumorskom i perifernom zdravom tkivu pluća u 50 bolesnika s NSCLC-om, od kojih 27 s AC-om i 23 sa SCC-om. Intenzitet citoplazmatskoga bojenja ocijenjen je od 0 do 3. Usporedbe između tkiva i podtipova provedene su Mann-Whitneyevim *U*-testom, a povezanost s kliničkim varijablama utvrđena je Spearmanovim koeficijentom korelacije ranga. Ekspresija CYP2E1 bila je značajno viša u tumorskom nego u zdravom tkivu u ukupnoj skupini s NSCLC-om te u obama histološkim podtipovima (p<0,05). Ekspresija CYP1A1 bila je viša u tkivima AC-a nego u zdravom tkivu i tkivima SCC-a (u oba slučaja p<0,05). Ekspresija GSTM1 bila je viša u tumorskom tkivu pacijenata s NSCLC-om, gledaju li se ukupno, a također je bila viša u tkivima SCC-a nego u normalnim tkivima i tkivima AC-a (p<0,05). Ekspresija GSTT1 bila je viša u tkivima AC-a nego SCC-a (p<0,05). Ekspresija CYP1B1 i GSTP1 bila je viša u obama tumorskim tkivima, ali ne značajno. Ukupno uzevši, izoenzimi CYP i GST pokazali su tkivno i podtipno ovisne obrasce ekspresije u NSCLC-u. Uočene razlike u CYP2E1, CYP1A1, GSTM1 i GSTT1 upućuju na to da se AC i SCC razlikuju u metabolizmu ksenobiotika i staničnoj obrani te da ti izoenzimi možda pridonose metaboličkim procesima povezanima s patogenezom NSCLC-a.

Xenobiotic-metabolising enzymes may influence the development of non-small cell lung cancer (NSCLC) by balancing the metabolic activation and detoxification of environmental carcinogens and other xenobiotics. Cytochrome P450 enzymes CYP1A1, CYP1B1, and CYP2E1 catalyse phase I oxidative reactions (1), whereas GSTM1, GSTT1, and GSTP1 mediate glutathione-dependent phase II conjugation of electrophilic compounds (2). Beyond carcinogen metabolism, these enzymes may also affect tumour behaviour and treatment response. Recent NSCLC studies have implicated CYP1A1-related pathways in tumour progression and cisplatin resistance and CYP1B1 in taxane resistance (3,4,5,6). A significant portion of recent studies directly related to the topic report heterogeneous CYP and GST protein expression in NSCLC (7,8,9,10,11), yet data on phase I and II enzymes in the same patient group or on tumour and normal tissues remain limited. To address these knowledge gaps, we compared the immunohistochemical expression of CYP1A1, CYP1B1, CYP2E1, GSTM1, GSTT1, and GSTP1 between tumour and matched peripheral normal tissues of NSCLC patients as well as between adenocarcinoma (AC) and squamous cell carcinoma (SCC) tissues. We believe this is important because treating NSCLC as a single group may obscure histological subtype-specific expression patterns.

## MATERIALS AND METHODS

This retrospective study was approved by the Clinical Research Ethics Committee of the Kırıkkale University Faculty of Medicine (approval No. 12/14 of 10 May 2012) and conducted in accordance with the Declaration of Helsinki. Due to the retrospective design, individual informed consent to tissue analysis was not required, and all study data were anonymised.

An a priori power analysis was performed using G*Power version 3.1 (Heinrich Heine University, Düsseldorf, Germany) for a predefined tumour-normal tissue comparison for each marker. Based on an effect size of d=0.5, an alpha level of 0.05, and a statistical power of 0.80, the minimum required sample size was 34 patients. The study included 50 patients with a histopathologically confirmed diagnosis of NSCLC who had not received chemotherapy or radiotherapy prior to biopsy. Paraffin blocks containing sufficient tissue for immunohistochemical examination of six different isoenzymes were selected by a pathologist from the Pathology Archive of the Atatürk Sanatorium Training and Research Hospital. Data on age, sex, smoking status, histological subtype, and tumour stage (using the tumour-node-metastasis, i.e. TNM system) at diagnosis were obtained from hospital records as described elsewhere (12, 13). Cases were not retrospectively re-staged according to a later edition of the TNM system. Serial 4 μm thick sections were taken from selected paraffin blocks for each of the six isoenzymes and placed on poly-L-lysine-coated slides. Immunohistochemical staining was evaluated separately in tumour tissue and in peripheral normal lung tissue areas distant from the tumour within the same paraffin blocks.

Sections taken separately for each isoenzyme were deparaffinised in xylene, rehydrated through a graded alcohol series, and then transferred to distilled water. Endogenous peroxidase activity was blocked by incubating the sections for 10 min in methanol (Merck KGaA, Darmstadt, Germany) containing 1 % H_2_O_2_, prepared from a 3 % hydrogen peroxide solution (Boster Biological Technology, Pleasanton, CA, USA). Sections were washed in distilled water and then subjected to heat-induced antigen retrieval in a pressure cooker containing 0.01 mol/L citrate buffer (pH 6.0; Boster Biological Technology) for 3 min. They were then transferred to tris-buffered saline (TBS) prepared in-house using 0.05 mol/L tris-HCl and 0.15 mol/L NaCl obtained from Merck KGaA (Darmstadt, Germany). To prevent non-specific background staining, the sections were incubated for 10 min at room temperature with the Super Block reagent supplied in the SensiTek HRP Anti-Polyvalent Lab Pack (SHP125, ScyTek Laboratories, Inc., Logan, UT, USA). The sections were then incubated overnight at 4 °C with the following primary antibodies: anti-CYP1A1 (1:50, sc-20772, Santa Cruz Biotechnology, Dallas, TX, USA); anti-CYP1B1 (1:400, ab33586, Abcam, Cambridge, UK); anti-CYP2E1 (1:300, PA1116, Boster Biological Technology); anti-GSTP (1:500, PA1040, Boster Biological Technology); anti-GSTM1 (1:100, ab244483, Abcam); and anti-GSTT1 (1:200, ab175418, Abcam). After a 15-min wash in TBS, the sections were sequentially incubated with the biotinylated link antibody and the streptavidin-horseradish peroxidase (HRP) complex supplied in the SensiTek HRP Anti-Polyvalent Lab Pack (SHP125, ScyTek Laboratories), according to the manufacturer's instructions. Peroxidase activity was visualised using a 3,3′-diaminobenzidine (DAB) chromogen kit (GBI Labs, Mukilteo, WA, USA), according to the manufacturer's instructions. The nuclei were counterstained with haematoxylin, after which the sections were dehydrated and mounted. Positive and negative controls were included in each staining run. For negative controls, the primary antibody was replaced with TBS. Stained sections were evaluated at 400× magnification using a Leica DM5000 B research microscope (Leica Microsystems, Wetzlar, Germany). Brown cytoplasmic staining was considered positive immunoreactivity. Staining intensity was scored independently by two observers blinded to patient information as 0 (no staining), 1 (weak staining), 2 (moderate staining), or 3 (strong staining) as described elsewhere (14,15,16). Disagreements between the observers were resolved by consensus. A staining score greater than 0 was considered positive protein expression.

### Statistical analysis

All statistical analyses were run on the MINITAB statistical software version 14.12.0 (Minitab Inc., State College, PA, USA). Patient age is presented as mean ± standard deviation and range, whereas categorical demographic and clinical variables are presented as number and percentage. Immunohistochemical staining scores were evaluated as ordinal data and presented as mean ± standard error of the mean (SEM).

To evaluate differences in protein expression scores between tumour and peripheral normal tissues and between adenocarcinoma and squamous cell carcinoma subtypes we used the Mann-Whitney *U* test, whereas Spearman's rank correlation analysis was used to evaluate associations between protein expression scores and patient age, sex, smoking status, and tumour stage. Because the analyses were exploratory, p values are reported without adjustment for multiple comparisons. All tests were two-sided, and p<0.05 was considered statistically significant.

## RESULTS AND DISCUSSION

Table 1 shows the characteristics of the 50 study patients with histopathologically confirmed NSCLC: 27 with AC and 23 with SCC.

**Table 1:** Baseline characteristics of 50 patients with non-small cell lung cancer

**Characteristics**		**Value (number and %)**
Sex		
	Male	45 (90)
	Female	5 (10)
Histology		
	Squamous Cell Carcinoma	23 (46)
	Adenocarcinoma	27 (54)
Stage at diagnosis		
	Stage I	20 (40)
	Stage II	17 (34)
	Stage III	13 (26)
Smoking Status		
	Never	11 (22)
	Current	39 (78)
Age (years)		
	Mean±SD (range)	58.52±8.07 (41–77)

### Expression patterns of CYP isoenzymes in NSCLC tissues

**Figure 1:**
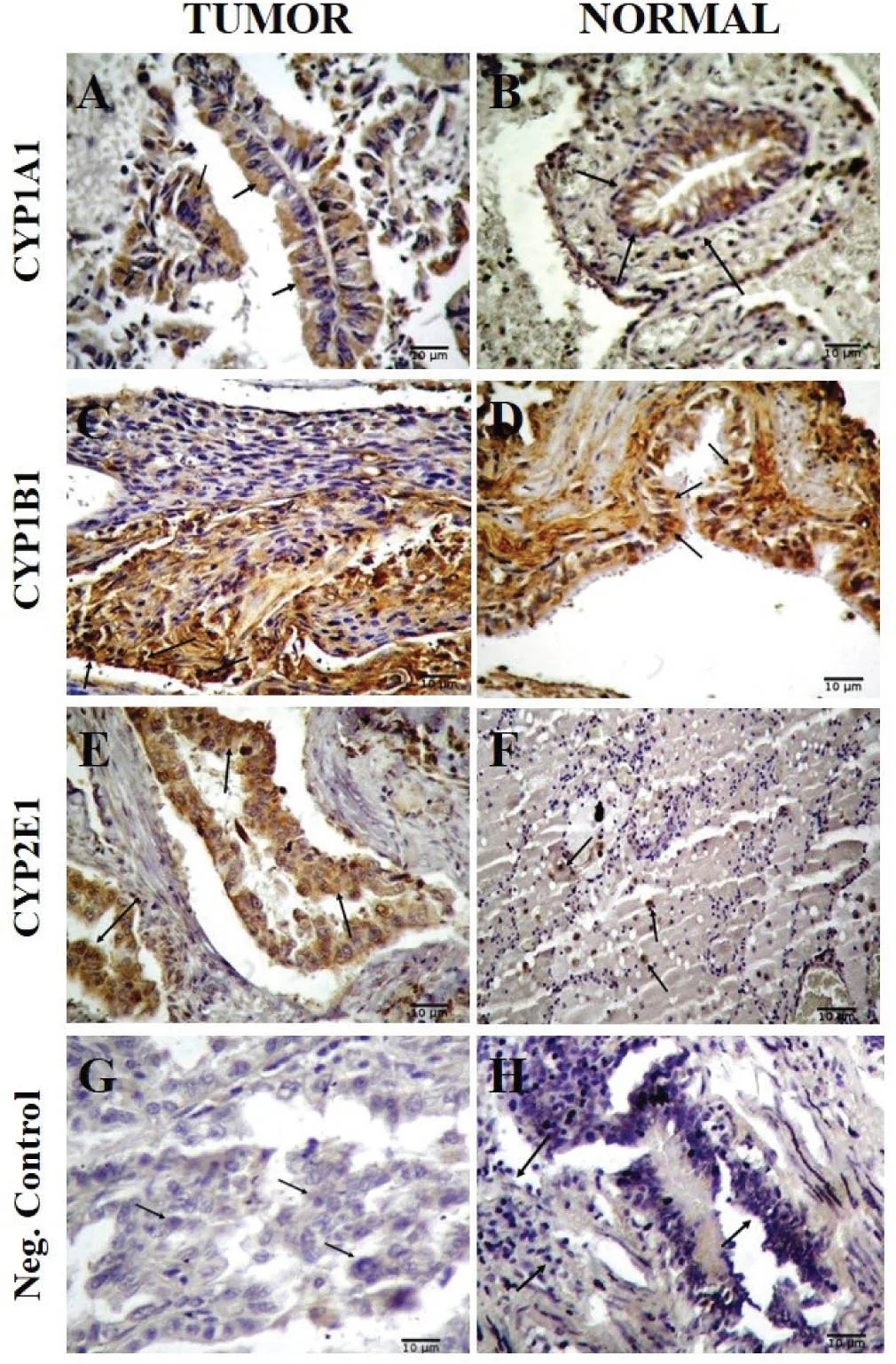
Immunohistochemical staining of CYP isoenzymes in tumour and peripheral normal lung tissues. A) CYP1A1 immunoreactivity in AC tumour cells; B) CYP1A1 immunoreactivity in bronchial epithelial cells of peripheral normal lung tissue; C) CYP1B1 immunoreactivity in SCC tumour cells; D) CYP1B1 immunoreactivity in bronchial epithelial cells of peripheral normal lung tissue; E) CYP2E1 immunoreactivity in AC tumour cells; F) CYP2E1 immunoreactivity in alveolar epithelial cells of peripheral normal lung tissue; and G–H) negative controls prepared without the primary antibody. Arrows in panels A–F indicate brown cytoplasmic immunoreactivity for the respective CYP isoenzyme. No specific brown cytoplasmic immunoreactivity was observed in the negative controls. Immunoreactivity was visualised using DAB, and nuclei were counterstained with haematoxylin. Images were acquired at 400× magnification. AC – adenocarcinoma; CYP – cytochrome P450; DAB – 3,3′-diaminobenzidine; Neg. Control – negative control; SCC – squamous cell carcinoma

Among the three CYP isoenzymes examined, CYP1A1 and CYP1B1 showed positive immunostaining in most NSCLC tumour samples, whereas CYP2E1 was detected less frequently. Positive immunostaining for CYP1A1, CYP1B1, and CYP2E1 was observed in tumour tissues from 46 (92 %), 48 (96 %), and 30 (60 %) patients, respectively (Table 2; Figure 1). Tumour expression was higher than that in the paired peripheral normal tissue in 21 patients (42 %) for CYP1A1, seven patients (14 %) for CYP1B1, and 27 patients (54 %) for CYP2E1 (Table 2).

**Table 2:** Number of patients with positive CYP protein expression in tumour tissues and the number of patients with higher CYP protein expression in tumour tissues than in paired peripheral normal tissues

**CYP isoenzymes**	**Expression category**	**NSCLC (N=50) n (%)**	**AC (N=27) n (%)**	**SCC (N=23) n (%)**
**CYP1A1**	Positive expression in tumour tissue	46 (92.00)	26 (96.30)	20 (86.96)
	Higher expression in tumour than in paired normal tissue	21 (42.00)	15 (55.56)	6 (26.09)
**CYP1B1**	Positive expression in tumour tissue	48 (96.00)	26 (96.30)	22 (95.65)
	Higher expression in tumour than in paired normal tissue	7 (14.00)	4 (14.81)	3 (13.04)
**CYP2E1**	Positive expression in tumour tissue	30 (60.00)	14 (51.85)	16 (69.57)
	Higher expression in tumour than in paired normal tissue	27 (54.00)	13 (48.15)	14 (60.87)

The staining intensity is graded as 0 (no staining), 1 (weak staining), 2 (moderate staining), and 3 (strong staining). Positive expression in tumour tissue is defined as a staining score greater than 0. Higher tumour expression is defined as a staining score in the tumour tissue greater than that in the paired peripheral normal tissue. Percentages are calculated using the total number of patients in each column as the denominator. AC – adenocarcinoma; N – number of patients; n – number of patients in the specific subgroup; NSCLC – non-small cell lung cancer; SCC – squamous cell carcinoma

**Figure 2:**
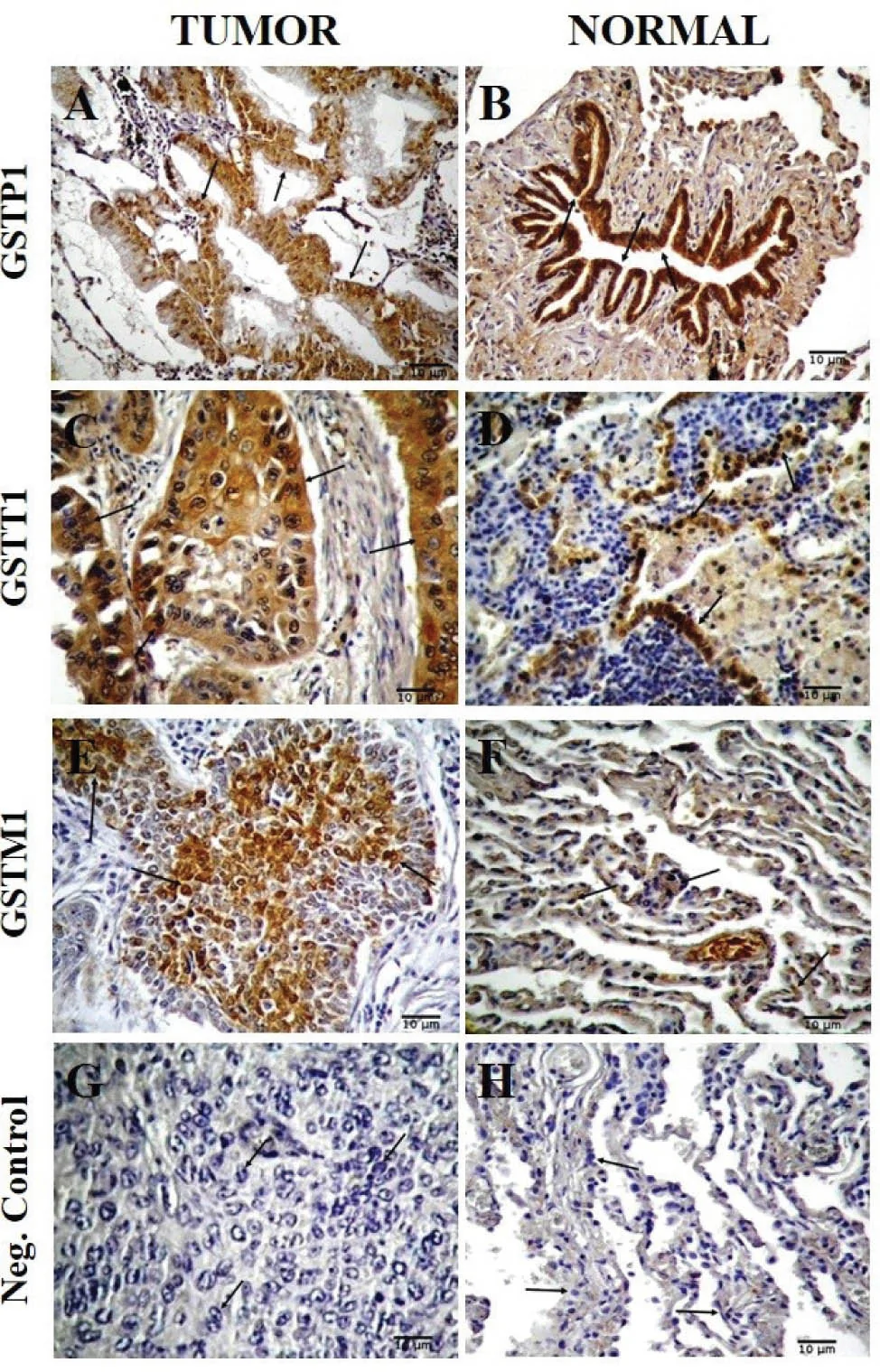
Immunohistochemical staining of GST isoenzymes in tumour and peripheral normal lung tissues. A) GSTP1 immunoreactivity in AC tumour cells; B) GSTP1 immunoreactivity in bronchial epithelial cells of peripheral normal lung tissue; C) GSTT1 immunoreactivity in AC tumour cells; D) GSTT1 immunoreactivity in alveolar epithelial cells of peripheral normal lung tissue; E) GSTM1 immunoreactivity in SCC tumour cells; F) GSTM1 immunoreactivity in alveolar epithelial cells of peripheral normal lung tissue; and G–H) negative controls prepared without the primary antibody. Arrows in panels A–F indicate brown cytoplasmic immunoreactivity for the respective GST isoenzyme. No specific brown cytoplasmic immunoreactivity was observed in the negative controls. Immunoreactivity was visualised using DAB, and nuclei were counterstained with haematoxylin. Images were acquired at 400× magnification. AC – adenocarcinoma; DAB – 3,3′-diaminobenzidine; GST – glutathione S-transferase; Neg. Control – negative control; SCC – squamous cell carcinoma.

When all patients with NSCLC were evaluated, CYP1A1 expression scores were numerically higher in tumour tissues than in paired normal tissues, although the difference was not statistically significant. CYP1B1 expression scores were high in both tumour and peripheral normal tissues and were numerically lower in tumour tissues, but the difference was not statistically significant. In contrast, CYP2E1 expression scores were significantly higher in tumour tissues than in paired peripheral normal tissues (p<0.001) (Table 3).

**Table 3:** Comparison of CYP protein expression scores between tumour and peripheral normal tissues and between histological subtypes in patients with NSCLC

		**CYP1A1**			**CYP1B1**			**CYP2E1**		
	**N**	**Tumour**	**Normal**	**T/N**	**Tumour**	**Normal**	**T/N**	**Tumour**	**Normal**	**T/N**
				p* value			p* value		p* value	
**NSCLC**	50	1.90±0.14^a^	1.54±0.16	1.23 0.1144	2.62±0.11	2.82±0.06	0.92 0.3521	0.84±0.13	0.12±0.05	7.00 **0.0001**
		(0–3)^b^	(0–3)		(0–3)	(1–3)		(0–3)	(0–2)	
**AC**	27	2.44±0.17	1.56±0.23	1.56 **0.0068**	2.67±0.15	2.85±0.07	0.93 0.7424	0.82±0.19	0.07±0.05	11.71 **0.0040**
		(0–3)	(0–3)		(0–3)	(2–3)		(0–3)	(0–1)	
**SCC**	23	1.26±0.16	1.52±0.22	0.83 0.5241	2.57±0.15	2.78±0.11	0.92 0.3337	0.87±0.16	0.17±0.10	5.12 **0.0013**
		(0–3)	(0–3)		(0–3)	(1–3)		(0–3)	(0–2)	
**AC/SCC**		1.94	1.03		1.04	1.03		0.94	0.40	
p** value		**0.0001**	0.9457		0.4192	0.8533		0.5143	0.7261	

T/N is the ratio of the mean expression score in tumour tissue to that in peripheral normal tissue within each group. AC/SCC is the ratio of the mean expression score in AC to that in SCC, calculated separately for tumour and peripheral normal tissues.

p^*^ value indicates the difference in expression scores between tumour and peripheral normal tissues in the overall NSCLC cohort and separately in the AC and SCC groups.

p^**^ value indicates the difference in expression scores between AC and SCC, calculated separately for tumour tissues (AC tumour vs SCC tumour) and peripheral normal tissues (AC normal vs SCC normal). Comparisons were performed using the Mann-Whitney *U* test, and p<0.05 is considered statistically significant

When the analyses were stratified by histological subtype, CYP1A1 and CYP2E1 expression scores were significantly higher in AC tumour tissues than in the paired peripheral normal tissues (p=0.0068 and p=0.0040, respectively). In the SCC group, a significant difference between the tumour and normal tissue was observed only for CYP2E1 (p=0.0013). AC tumours had significantly higher CYP1A1 protein expression (p=0.0001) when AC and SCC tumours were directly compared. There were no statistically significant differences in CYP1B1 or CYP2E1 expression between the two histological subtypes (Table 3).

Furthermore, no statistically significant associations were observed between the CYP protein expression and patient age, sex, smoking status, or tumour stage.

Previous studies have reported inter-patient and tissue-specific variability in CYP isoenzyme expression in human NSCLC. Toussaint et al. (8) examined paired tumour and non-tumour lung tissues from 12 patients with NSCLC and reported CYP1A1/1A2 protein levels to be about three times higher in non-tumour than in tumour tissues, whereas the other CYP isoenzymes examined were not detectable. The higher frequency of CYP1A1 immunostaining observed in our study may reflect differences in sample size, tissue processing, antibody specificity, analytical methodology, and criteria used to define positive expression. Spivack et al. (11) also reported gene- and tissue-specific differences in the expression of phase I and II xenobiotic-metabolising enzymes between paired tumour and non-tumour lung tissues. Oyama et al. (7) reported that CYP1A1, CYP2A6, CYP2E1, and CYP3A proteins were detected more frequently in AC than in SCC.

In our study, CYP1A1 was detected in most tumour samples, but there was no significant difference between the tumour and normal tissue in the overall NSCLC group. In contrast, CYP1A1 expression was significantly higher in AC tumours than in both paired normal tissues and SCC tumours. Thus, the main CYP1A1 finding was not a general tumour-associated increase, but the difference is mainly associated with the AC subtype.

Experimental studies provide some biological context for this finding. Wang et al. (3) reported increased A-to-I RNA editing of *CYP1A1* in NSCLC tissues and showed that the RNA-edited variant promoted tumour progression and resistance to oxidative stress through PI3K/Akt-dependent activation of HO-1. Kang et al. (17) also showed that CYP1A1 expression could be modified by metabolic conditions in experimental lung cancer models and that its induction involved AhR-dependent regulation. These findings support the biological relevance of CYP1A1 regulation in lung cancer, but our study did not investigate the mechanisms underlying the AC-specific protein expression difference.

Although CYP1B1 was the most frequently detected CYP isoenzyme in our study, its expression was not significantly higher in tumour than in paired normal tissues. Lin et al. (9) reported an association between CYP1B1 and aryl hydrocarbon receptor (AhR) expression in human NSCLC tissues, suggesting that the regulation of these two proteins may be coordinated. Słowikowski et al. (18), who used RT-qPCR and Western blotting in matched tumour and histopathologically unaltered lung samples from 76 patients with NSCLC, reported lower *CYP1B1* mRNA and CYP1B1 protein levels in tumour tissue. This finding is consistent with our results, as CYP1B1 expression was also numerically lower in tumour tissue, although the difference between tumour and paired normal tissue was not statistically significant. At the protein level, the difference in the magnitude and statistical significance of the findings may partly reflect methodological differences, since immunohistochemistry provides semi-quantitative and spatial information, whereas Western blotting measures total protein abundance in tissue homogenates.

CYP2E1 demonstrated the most pronounced and consistent difference between tumour and normal tissue, despite being positive in fewer tumour samples than CYP1A1 and CYP1B1. Its expression scores were significantly higher in both AC and SCC tumours than in their paired normal tissues, suggesting that the tumour-associated increase was not restricted to a single histological subtype. Consistent with our subtype comparison, Kivistö et al. (10) reported immunohistochemical expression of CYP2E1 in both primary lung carcinomas and normal bronchial tissues, with no significant difference between AC and SCC tumours.

Jia et al. (19) reported higher CYP2E1 expression in peritumoural tissues from patients with NSCLC than in normal lung tissues. In experimental models in the same study, genetic deletion of *Cyp2e1* and pharmacological inhibition of CYP2E1 with Q11 suppressed lung tumour growth, with the effect of Q11 attributed mainly to modulation of macrophage polarisation in the inflammatory tumour microenvironment. However, instead of comparing tumour and peripheral normal tissue like we did, Jia et al. compared peritumoural tissue with normal lung tissue. Their findings are therefore not directly comparable with ours but suggest that CYP2E1-related changes in NSCLC may also involve the tumour microenvironment (19).

Overall, CYP isoenzyme expression in NSCLC was heterogeneous and varied according to isoenzyme and histological subtype.

### Expression patterns of GST isoenzymes in NSCLC tissues

GSTP1 was the most frequently detected GST isoenzyme in NSCLC tumour tissues, followed by GSTT1. Positive immunostaining for GSTP1, GSTT1, and GSTM1 was observed in tumour tissues from 48 (96 %), 39 (78 %), and 19 (38 %) patients, respectively. Expression in tumour tissue was higher than in matched peripheral normal tissue in 15 (30 %) patients for GSTP1, 16 (32 %) for GSTT1, and 17 (34 %) for GSTM1 (Table 4).

**Table 4:** Number of patients with positive GST protein expression in tumour tissues and the number of patients with higher GST protein expression in tumour tissues than in paired peripheral normal tissues

**GST isoenzyme**	**Expression category**	**NSCLC (N=50) n (%)**	**AC (N=27) n (%)**	**SCC (N=23) n (%)**
**GSTP1**	Positive expression in tumour tissue	48 (96.00)	25 (92.59)	23 (100.00)
	Higher expression in tumour than in paired normal tissue	15 (30.00)	8 (29.63)	7 (30.43)
**GSTT1**	Positive expression in tumour tissue	39 (78.00)	24 (88.89)	15 (65.22)
	Higher expression in tumour than in paired normal tissue	16 (32.00)	10 (37.04)	6 (26.09)
**GSTM1**	Positive expression in tumour tissue	19 (38.00)	6 (22.22)	13 (56.52)
	Higher expression in tumour than in paired normal tissue	17 (34.00)	5 (18.52)	12 (52.17)

The staining intensity is graded as 0 (no staining), 1 (weak staining), 2 (moderate staining), and 3 (strong staining). Positive expression in tumour tissue is defined as a staining score greater than 0. Higher tumour expression is defined as a staining score in the tumour tissue greater than that in the paired peripheral normal tissue. Percentages are calculated using the total number of patients in each column as the denominator. AC – adenocarcinoma; N – number of patients; n – number of patients in the specific subgroup; NSCLC – non-small cell lung cancer; SCC – squamous cell carcinoma

When the overall NSCLC group was assessed, only GSTM1 expression differed significantly between tumour and matched normal tissues, with higher expression in tumour tissue (p=0.0122) (Table 5). This indicates that, among the three GST isoenzymes examined, GSTM1 showed the clearest tumour-associated expression difference in the overall patient group.

**Table 5:** Comparison of GST protein expression scores between tumour and peripheral normal tissues and between histological subtypes in patients with NSCLC

		**GSTP1**			**GSTM1**			**GSTT1**		
	**N**	**Tumour**	**Normal**	**T/N**	**Tumour**	**Normal**	**T/N**	**Tumour**	**Normal**	**T/N**
				p* value			p* value			p* value
**NSCLC**	50	2.44±0.12^a^	2.22±0.12	1.09 0.2121	0.70±0.15	0.14±0.06	5.00 **0.0122**	1.54±0.16	1.48±0.18	1.08 0.7459
		(0–3)^b^	(1–3)		(0–3)	(0–2)		(0–3)	(0–3)	
**AC**	27	2.33±0.19	2.15±0.18	1.08 0.4312	0.41±0.17	0.19±0.11	2.15 0.4835	2.11±0.20	1.67±0.26	1.26 0.2796
		(0–3)	(1–3)		(0–3)	(0–2)		(0–3)	(0–3)	
**SCC**	23	2.57±0.14	2.30±0.17	1.11 0.3448	1.04±0.23	0.09±0.06	11.60 **0.0032**	0.87±0.18	1.26±0.26	0.69 0.4485
		(1–3)	(1–3)		(0–3)	(0–1)		(0–3)	(0–3)	
**AC/SCC**		0.91	0.93		0.39	2.11		2.43	1.33	
p** value		0.5724	0.6060		**0.0373**	0.8609		**0.0003**	0.3304	

T/N is the ratio of the mean expression score in tumour tissue to that in peripheral normal tissue within each group. AC/SCC is the ratio of the mean expression score in AC to that in SCC, calculated separately for tumour and peripheral normal tissues.

p^*^ value indicates the difference in expression scores between tumour and peripheral normal tissues in the overall NSCLC cohort and separately in the AC and SCC groups.

p^**^ value indicates the difference in expression scores between AC and SCC, calculated separately for tumour tissues (AC tumour vs SCC tumour) and peripheral normal tissues (AC normal vs SCC normal). Comparisons were performed using the Mann-Whitney *U* test, and p<0.05 is considered statistically significant

When stratified by histological subtype, GSTM1 expression was significantly higher in SCC tumours than in matched normal tissues (p=0.0032) (Table 5), suggesting that the overall tumour-associated increase in GSTM1 was driven mainly by the SCC subgroup. In direct comparisons between histological subtypes, GSTM1 expression was also significantly higher in SCC than in AC tumours (p=0.0373), whereas GSTT1 expression was significantly higher in AC than in SCC tumours (p=0.0003) (Table 5).

GSTP1 was widely expressed in both tumour and matched peripheral normal tissues, but our findings did not show a significant tumour- or histological subtype-specific increase. Thus, its high expression in NSCLC appears to reflect widespread expression in lung tissue from these patients rather than selective upregulation in tumour tissue.

GSTP1 has functions beyond glutathione conjugation and has been implicated in cellular redox and stress responses and in the regulation of JNK-dependent apoptosis (20, 21). In lung adenocarcinoma models, GSTP1 has also been associated with oxidative stress adaptation, cancer stem cell properties, and resistance to tyrosine kinase inhibitors, while GSTP1 inhibition increased treatment sensitivity (22). These findings provide a biological context for the widespread GSTP1 expression observed in our study, yet we did not examine the underlying pathways.

Clinical studies have associated GSTP1 expression with chemotherapy response and drug resistance in NSCLC (23, 24). As the tissues in our study were obtained before chemotherapy, the observed GSTP1 expression represents pre-treatment expression rather than a treatment-induced change. However, we did not assess GSTP1 enzyme activity, related signalling pathways, or treatment response, and therefore cannot determine whether GSTP1 expression in our patients contributed to drug resistance.

GSTM1 showed the lowest staining frequency among the GST isoenzymes, but its tumour-associated increase was particularly evident in SCC. Spivack et al. (11) reported that *GSTM1* mRNA expression was infrequent in human lung tissue. Despite the different analytical approach, this is consistent with the relatively low frequency of GSTM1 positivity in our cohort. Taken together, the low overall frequency of GSTM1 expression and its preferential increase in SCC suggest that GSTM1 may characterise a subset of NSCLC tumours rather than a general tumour-associated expression.

The principal GSTT1 finding was higher expression in AC than in SCC tumours, rather than a general tumour-associated increase (Table 5). Spivack et al. (11) reported GSTT1 expression in both tumour and non-tumour lung tissues, and Crawford et al. (25) found no significant difference in *GSTT1* transcript levels between the compared bronchial epithelial groups. These findings are consistent with ours but do not explain higher GSTT1 expression observed in AC than in SCC tumours. We believe that it may indicate differences in xenobiotic metabolism or cellular defence between these two subtypes. However, we did not evaluate GSTT1 enzyme activity and related molecular pathways to be able to establish the biological basis of this difference.

As with CYP isoenzymes, GST protein expression was not significantly associated with patient age, sex, smoking status, or tumour stage.

Overall, our findings show that CYP and GST isoenzymes do not have a common or unidirectional expression pattern in NSCLC. Instead, the observed differences depended on both the specific isoenzyme and histological subtype, with CYP1A1 and GSTT1 differences more evident in AC and GSTM1 in SCC, whereas CYP2E1 showed a tumour-associated increase in both subtypes.

## CONCLUSION

One strength of this study is the simultaneous evaluation of phase I and II xenobiotic-metabolising enzymes at the protein level in the same group of patients with NSCLC. By comparing tumour tissue with matched peripheral normal tissue from the same patient we reduced the influence of inter-individual variability. By subtyping NSCLC, we could also establish histological differences in CYP and GST expression between AC and SCC. In addition, immunohistochemical scoring by two observers blinded to patient information rendered the assessment more reliable.

However, our study has several limitations. It is retrospective by design and included a relatively small patient group from a single location. Although the power analysis indicated an adequate sample size for the overall tumour v normal tissue comparisons, the AC and SCC subgroup analyses may have lowered its statistical power.

Another design limitation is that protein expression was assessed semi-quantitatively, by immunohistochemistry, rather than by an independent quantitative protein method, nor did we measure enzyme activity.

In addition, we had no detailed information on the duration and intensity of smoking exposure, comorbidities, medications, and other environmental or clinical factors to account for their effects on CYP and GST expression.

Furthermore, as we obtained normal tissues from the same paraffin blocks taken from patients with NSCLC rather than from individuals without cancer, possible site-specific effects in apparently normal tissues cannot be completely ruled out.

Finally, because relevant signalling pathways, oxidative stress and apoptosis markers, and patient treatment responses were not evaluated, the observed differences in protein expression cannot support direct mechanistic, prognostic, or causal conclusions regarding treatment resistance. However, evaluating CYP and GST proteins in conjunction with histological subtype and matched tissue differences may help prioritise candidate isoenzymes and patient subgroups for future mechanistic and treatment-response studies. Before that, our findings should be validated in larger studies with independent cohorts that will quantify the proteins and enzyme activity and determine molecular pathways and clinical outcomes.

## Abbreviations

AC: adenocarcinoma
AhR: aryl hydrocarbon receptor
Akt: protein kinase B
CYP: cytochrome P450
CYP1A1: cytochrome P450 family 1 subfamily A member 1
CYP1B1: cytochrome P450 family 1 subfamily B member 1
CYP2A6: cytochrome P450 family 2 subfamily A member 6
CYP2E1: cytochrome P450 family 2 subfamily E member 1
CYP3A: cytochrome P450 family 3 subfamily A
DAB: 3,3′-diaminobenzidine
GST: glutathione S-transferase
GSTM1: glutathione S-transferase mu 1
GSTP: glutathione S-transferase pi
GSTP1: glutathione S-transferase pi 1
GSTT1: glutathione S-transferase theta 1
HO-1: haem oxygenase 1
HRP: horseradish peroxidase
JNK: c-Jun N-terminal kinase
NSCLC: non-small cell lung cancer
PI3K: phosphoinositide 3-kinase
RNA: ribonucleic acid
RT-qPCR: reverse transcription quantitative polymerase chain reaction
SCC: squamous cell carcinoma
SD: standard deviation
SEM: standard error of the mean
TBS: tris-buffered saline
TNM: tumour–node–metastasis
T/N: tumour-to-peripheral normal tissue mean expression ratio
